# Raised Plasma Neurofilament Light Protein Levels Are Associated with Abnormal MRI Outcomes in Newborns Undergoing Therapeutic Hypothermia

**DOI:** 10.3389/fneur.2018.00086

**Published:** 2018-03-05

**Authors:** Divyen K. Shah, Vennila Ponnusamy, Jane Evanson, Olga Kapellou, Georgia Ekitzidou, Neelam Gupta, Paul Clarke, Adina T. Michael-Titus, Ping K. Yip

**Affiliations:** ^1^The Royal London Hospital, Barts Health NHS Trust, London, United Kingdom; ^2^The Centre for Neuroscience and Trauma, Barts and The London School of Medicine and Dentistry, Blizard Institute, Queen Mary University of London, London, United Kingdom; ^3^Centre for Genomics and Child Health, Barts and The London School of Medicine and Dentistry, Blizard Institute, Queen Mary University of London, London, United Kingdom; ^4^Ashford and St. Peter’s Hospitals NHS Foundation Trust, Chertsey, United Kingdom; ^5^Homerton University Hospital NHS Foundation Trust, London, United Kingdom; ^6^University Hospital Southampton, Southampton, United Kingdom; ^7^Norfolk and Norwich University Hospitals NHS Foundation Trust, Norwich, United Kingdom

**Keywords:** neurofilament proteins, hypoxic-ischemic encephalopathy, therapeutic hypothermia, MRI imaging, neuroprotection, biomarkers

## Abstract

**Aims and hypothesis:**

Hypoxic-ischemic encephalopathy (HIE) remains an important cause of death and disability in newborns. Mild therapeutic hypothermia (TH) is safe and effective; however, there are no tissue biomarkers available at the bedside to select babies for treatment. The aim of this study was to show that it is feasible to study plasma neurofilament light (NfL) levels from newborns and to evaluate their temporal course. Hypothesis: Raised plasma NFL protein levels from newborns who undergo TH after HIE are associated with abnormal MRI outcomes.

**Methods:**

Between February 2014 and January 2016, term newborns with HIE treated with TH for 72 h had plasma samples taken at three time points: (i) after the infant had reached target temperature, (ii) prior to commencing rewarming, and (iii) after completing rewarming. Infants with mild HIE who did not receive TH had a single specimen taken. NfL protein was analyzed using an enzyme-linked immunosorbent assay.

**Results:**

Twenty-six newborns with moderate–severe HIE treated with TH were studied. Half of these had cerebral MRI predictive of an unfavorable outcome. Plasma NfL levels were significantly higher in the TH group with unfavorable outcome (median age 18 h) compared to levels from both the mild HIE group and TH group with favorable outcome (*F* = 25.83, *p* < 0.0001). Newborns who had MRIs predictive of unfavorable outcome had significantly higher NfL levels compared to those with favorable outcomes, at all three time points (mixed models, *F* = 27.63, *p* < 0.001). A cutoff NfL level >29 pg/mL at 24 h is predictive of an unfavorable outcome [sensitivity 77%, specificity 69%, positive predictive value (PPV) 67%, negative predictive value (NPV) 72%] with increasing predictive value until after rewarming (sensitivity 92%, specificity 92%, PPV 92%, NPV 86%).

**Interpretation of research:**

Plasma NfL protein levels may be a useful biomarker of unfavorable MRI outcomes in newborns with moderate–severe HIE and may assist in selecting newborns for adjunctive neuroprotective interventions. Larger studies with NfL testing at earlier time points are required.

## Introduction

Perinatal asphyxia in the newborn remains an important cause of death and of disability in survivors. The resulting brain dysfunction and neonatal encephalopathy occurs in up to 4 per 1,000 live births in western countries (1, 2).

Mild therapeutic hypothermia (TH) treatment started within 6 h after birth, has been shown to be safe and effective in reducing death and disability, with numbers needed to treat (NNT) of 7–9 (3, 4). However, many babies go on to have adverse outcomes despite TH (5, 6). At present, empirical criteria are used to select babies for TH (7). Various compounds have been studied in the plasma, urine, and cerebrospinal fluid (CSF) as potential early biomarkers of CNS injury (8). There are no established blood biomarkers available to assist early cot side identification of babies most likely to benefit from neuroprotection. As a result, a number of babies with hypoxic-ischemic encephalopathy (HIE) who do not fulfill empirical criteria and who do not receive TH are noted to have adverse outcomes (9).

Neurofilaments, a group of intermediate sized filamentous proteins, are the most abundant cytoskeletal component, found predominantly in the myelinated axons of the central nervous system (10). They contribute to axonal volume and the radial diameter and hence are important for efficient transmission of action potentials. The neurofilament light (NfL) protein subunit is primarily expressed in large caliber myelinated axons.

When neurons are damaged, the NfL protein is released into the CSF and blood (10). An increased NfL protein level in CSF and blood is associated with the severity of neuropathology in adults with diseases including amyotrophic lateral sclerosis (ALS) (11), spinal cord injury (12), and multiple sclerosis (13). In asphyxiated newborns, neurofilament levels have been shown to be raised in CSF (14) and Toorell et al. (15) have recently reported raised levels in cord blood from asphyxiated newborns when compared to term controls. To date, there are no studies reporting the feasibility of studying plasma NfL levels in newborns with HIE who undergo TH and in the evaluation of their temporal course. We hypothesized that plasma NfL levels are significantly raised in newborns with cerebral MRI predictive of unfavorable neurodevelopmental outcomes.

If NfL is shown to be a reliable biomarker, then the selection of newborns for TH will be more effective with objective targeting of babies who will benefit most. The injury cascade affecting the newborn brain after hypoxia-ischemia continues for hours and days after the initial insult, with multiple mechanisms being implicated and hence various potential adjunctive neuroprotection targets could be considered (16). If reliable, NfL may prove to be a suitable biomarker of neuronal injury and useful for the evaluation of additional neuroprotective interventions.

## Materials and Methods

### Study Population

Between January 2014 and January 2016, newborns were recruited into the Brain Injury Biomarkers in Newborns study from five tertiary neonatal centers; the Royal London Hospital, Homerton University Hospital, Ashford and St. Peter’s Hospitals NHS Foundation Trust, University Hospital Southampton, and Norfolk and Norwich University Hospital. Babies were enrolled after written consent was obtained from the parents and this study had research ethics committee approval (REC reference: 13/LO/17380).

Two categories of babies were included: (1) 11 consecutively recruited term newborns admitted to the neonatal units with acidosis and/or mild HIE who did not fulfill standard criteria for TH and were managed conservatively and (2) babies who fulfilled standard cooling criteria (UK TOBY Cooling Register Clinician’s Handbook) who underwent TH and had cerebral MR imaging. Of the latter group, the first 13 recruited with MR imaging predictive of favorable outcome and the first 13 with MR imaging predictive of unfavorable outcomes were included in the study. Exclusion criteria included death before an MRI was obtained (TH group), images degraded by significant motion artifact, major congenital anomaly, or a primary diagnosis of an inborn error of metabolism.

Blood from the babies with mild HIE who did not undergo TH was obtained at a single time point. Samples from babies who received TH were obtained at three time points in the first week after birth: (i) after the target temperature of 33–34°C was reached (S1), (ii) prior to rewarming (S2), and (iii) after rewarming was completed (S3).

### Preparation and Analysis of Plasma Samples

Collected blood samples were transferred into spray-coated K_2_EDTA tubes (Fisher Scientific Ltd., Loughborough, UK), then centrifuged at 15,000 × *g* for 10 min to separate plasma, which was stored at −80°C until analysis.

A commercially available enzyme-linked immunosorbent assay (ELISA) (UD51001, IBL-International GmbH) was used to quantify NfL. Bovine NfL standards ranging from 0 to 10,000 pg/mL and 100 µL of diluted samples from each newborn (50 µL of neat plasma sample diluted with 160 µL sample diluent) were run in duplicate, following the manufacturer’s instructions. The optical density of each sample was determined at a wavelength of 450 nm. NfL concentrations were calculated using the inverse formula: *x* = *c*(((*a* − *d*)/(*y* − *d*)) − 1)^1/^*^b^* provided by the online ELISA software (http://elisaanalysis.com). This study was carried out with three NfL ELISA plates from different batches at different times, to ensure the test was not affected by variability between plates. Each ELISA plate contained samples from all three groups and from different time points. Each plate was processed at a different time. The mean of the two results was reported. A coefficient of variation of up to 10% was accepted.

### Outcomes

Infants who underwent TH had cerebral MR imaging. MRI was performed at local centers with conventional T1-weighted and T2-weighted sequences at 1.5 T. MR images were independently rated by a neuroradiologist (JE) and a neonatologist with imaging expertise (OK) who were blinded to clinical information. All images were of sufficient quality to be reportable by each rater. Patterns of MRI injury were classified into two groups using the system described by Rutherford et al. (17), which has prognostic value in infants who have undergone TH. Infants with an unfavorable outcome had a severe pattern of injury including reversed or abnormal signal intensity bilaterally on T1- and/or T2-weighted sequences in the posterior limb of the internal capsule (PLIC); multifocal or widespread abnormal signal intensity in the basal ganglia and thalami (BGT); and severe widespread white matter (WM) lesions including infarction, hemorrhage and long T1 and T2. Infants with MRIs predictive of a favorable outcome had either normal images or less severe patterns of injury that are associated with normal or only mildly abnormal neurodevelopmental outcomes. Consensus was reached in cases of disagreement.

Infants who did not qualify for TH were classified as having a favorable outcome if they had a normal neurologic examination at 24 h of age and were discharged home on suck feeding. These infants did not have MR imaging.

### Statistical Analysis

Statistical analysis was carried out using SPSS (version 22). Continuous variables between groups were compared using ANOVA and proportions were compared using Pearson’s chi-square statistic. NfL levels for S1 were positively skewed and were log transformed prior to analysis with ANOVA. Receiver operated characteristics (ROC) curves were created comparing the mild HIE group with the TH unfavorable outcome group at S1 and also comparing the TH favorable with the unfavorable outcome group at all three time points S1, S2, and S3. For each ROC curve, a cutoff level was obtained where the sensitivity + specificity was at its maximum. All graphs and ROC were created using GraphPad Prism (version 6.0). *p* < 0.05 was used as the level of statistical significance.

## Results

The perinatal characteristics of the newborns studied are described in Table 1. Babies in the unfavorable outcome group were more likely to have had a lower 10-min Apgar score, and to have had chest compressions at resuscitation, anticonvulsants, and inotropic support.

**Table 1:** Comparison of the clinical characteristics of the three groups of babies studied.

	MildNo therapeutic hypothermia (TH)	Mod-severeTH favorable	Mod-severeTH unfavorable	*p*-Value
*n*	11	13	13	
Male	5/11	9/13	9/13	0.39
Birth weight (g)	3,360 (2,971, 3,987)	3,546 (3,045, 3,880)	3,440 (2,800, 3,770)	0.70
PMA	40 (39, 41.29)	39.57 (38.86, 41)	40.14 (38, 41)	0.73
10-min Apgar	9 (8,9)	6 (4,7)	5 (4,6)	0.001
Chest compressions	0/9	3/10	7/11	0.035
Resuscitation cardiac drugs	0/9	2/12	5/11	0.13
Worst pH in first hour	6.96 (6.91,7.03)	6.90 (6.71, 7.00)	6.87 (6.77,6.94)	0.27
Worst base deficit in first hour	−15 (−12, −8)	−17 (−13, −21)	−20 (−18, −23)	0.14
Maternal pyrexia	2/10	0/12	0/12	0.22
Chorioamnionitis	0/9	0/13	0/12	0.52
Sentinel event	1/11	4/13	3/13	0.25
Meconium aspiration	0/10	1/13	2/12	0.41
Blood glucose (<2.6 mmol/l)	0/8	0/8	0/9	0.83
Positive blood culture	0/10	0/13	1/13 (CONS*)	0.38
Clinical seizures	0/10	8/13	12/13	<0.001
Anticonvulsants given	0/10	7/13	10/12	<0.001
Inotropes used	0/11	2/13	9/12	<0.001
Age at MRI scan (days)	–	8 (7, 9)	9 (8, 12)	0.56

*Data in brackets represent median interquartile range*.

CONS*, coagulase negative staphylococci.

### MRI Outcome

**Figure 1:**
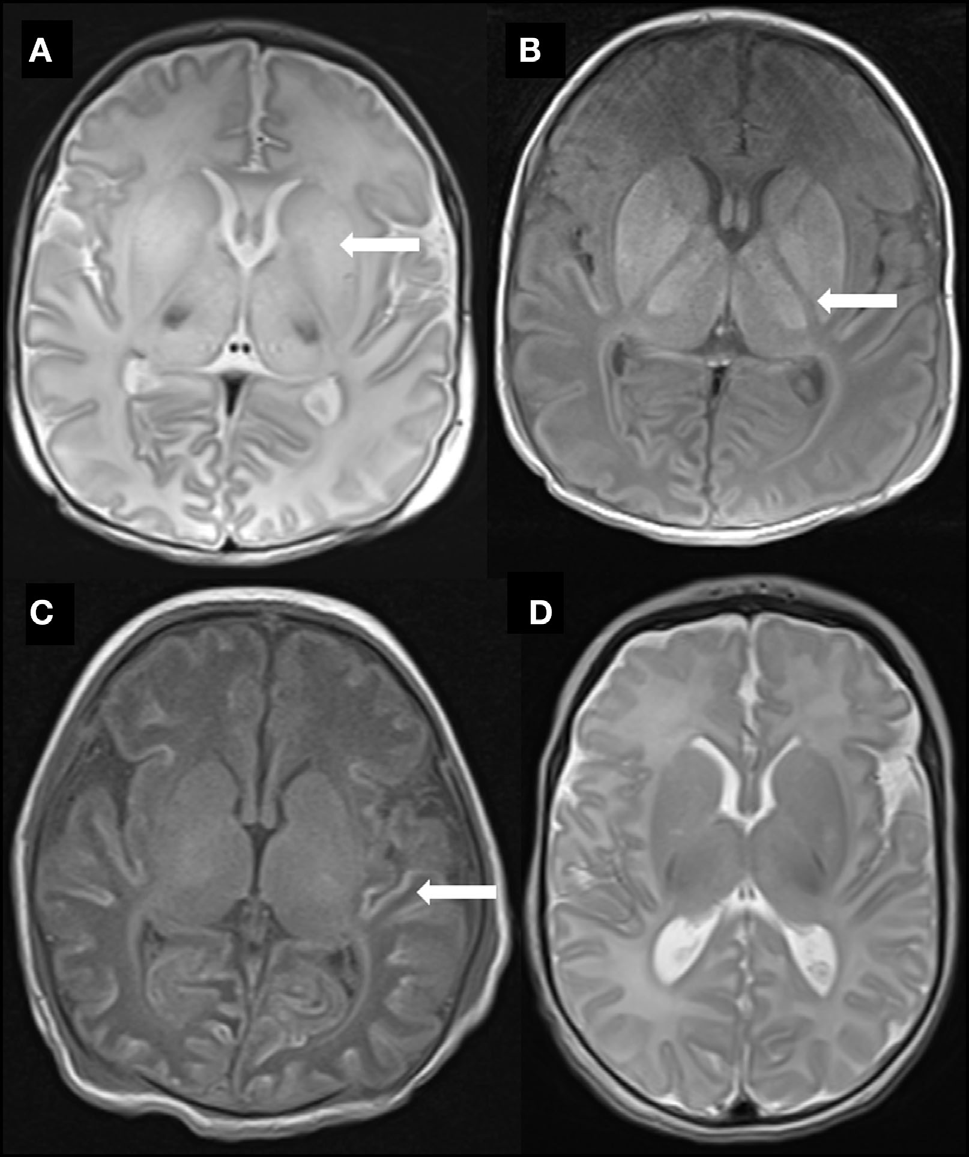
Representative MR images of study babies. **(A)** Diffuse high T2 signal throughout the basal ganglia (arrow) from baby 3. **(B)** Abnormal high T1 signal throughout the basal ganglia and thalami as well as complete loss of signal in the posterior limb of internal capsule (arrow) also from baby 3. **(C)** Widespread T1 high signal (arrow) in cortex (“cortical highlighting”) from baby 6. **(D)** Normal signal throughout the basal ganglia in T2-weighted image from baby 23.

The 26 babies who underwent TH all had MRI performed at a median age of 9 [interquartile range (IQR) 8, 11] days. There was no statistical difference in age at MRI between the 13 babies with MRI predictive of favorable outcome and the other 13 with MRI predictive of unfavorable outcome (*F* = 0.89, *p* = 0.56) (Table 1). Of the 13 infants with images predictive of unfavorable outcome, six had a combination of subcortical WM and BGT abnormalities, five had predominantly BGT abnormalities, and two had predominantly subcortical WM abnormalities (Table 2). Representative images are shown in Figure 1. Overall scores for the MR images of the 26 babies who underwent TH showed 96% inter-rater agreement.

**Table 2:** Patients arranged in descending rank order of neurofilament light levels at S3 in relation to MRI findings and neurological exam at discharge. The latter is classified primarily in terms of abnormality of tone.

	S1 (pg/mL)	S2 (pg/mL)	S3 (pg/mL)	Age at peak (h)	Electrical seizures (Y/N)	PLIC	BGT	WM	Cortex	Composite MRI Group	Sentinel event (Y/N)	Comment	Neurologic exam prior to discharge
1	2,506	2,255	4,698	100	Y	Loss	Severe	Moderate	Normal	U	N		Severe, died after 18 months
2	1,999	1,615	1,993	18	N	Equivocal	Normal	Severe	Moderate	U	Y	Cord prolapse	Mild
3	0	189	1,626	124	Y	Loss	Severe	Severe	Severe	U	N		Severe, died after 18 months
4	70	423	1,014	114	N	Loss	Severe	Mild	Mild	U	Y	Breech, head entrapment	Moderate, tube feeds
5	1,046	1,512	874	31	Y	Normal	Moderate	Normal	Normal	U	N		Normal
6	516	n/a	849	96	N	Equivocal	Normal	Severe	Severe	U	Y	Placental abruption	Severe, tube feeds
7	413	381	844	118	Y	Normal	Moderate	Severe	Moderate	U	N		Moderate, tube feeds
8	29	225	674	95	N	Loss	Severe	Severe	Severe	U	Y	Shoulder dystocia	Severe, tube feeds
9	0	n/a	563	94	Y	Equivocal	Moderate	Mild	Moderate	U	N		Moderate, tube feeds
10	0	0	511	96	n/a	Loss	Moderate	Mild	Normal	U	N		Severe, tube feeds
11	0	294	506	100	Y	Normal	Mild	Mild	Normal	F	N		Normal
12	681	419	490	18	N	Loss	Severe	Normal	Moderate	U	N		Normal
13	141	321	403	115	N	Normal	Normal	Normal	Normal	F	Y	Placental abruption	Normal
14	99	577	381	67	N	Normal	Normal	Normal	Normal	F	Y	Head entrapment	Normal
15	28	77	336	101	N	Normal	Normal	Mild	Mild	F	Y	Shoulder dystocia	Normal
16	173	159	334	101	N	Loss	Severe	Moderate	Mild	U	N		Normal
17	277	457	324	62	N	Normal	Normal	Normal	Normal	F	N		Mild
18	166	160	242	86	Y	Normal	Mild	Normal	Normal	F	N		Normal
19	0	71	164	108	n/a	Normal	Normal	Mild	Normal	F	N		Normal
20	148	172	151	48	Y	Loss	Moderate	Severe	Normal	U	N		Moderate, tube feeds
21	0	41	143	115	n/a	Normal	Normal	Normal	Normal	F	N		Normal
22	0	0	136	114	Y	Normal	Normal	Moderate	Normal	F	N		Normal
23	0	0	59	102	N	Normal	Normal	Normal	Normal	F	N		Normal
24	0	0	8	-	n/a	Normal	Normal	Normal	Normal	F	N		Normal
25	0	0	0	-	Y	Normal	Normal	Mild	Mild	F	N		Normal
26	0	0	0	-	N	Normal	Normal	Normal	Normal	F	Y	Uterine rupture	Normal

*PLIC, posterior limb of internal capsule; BGT, basal ganglia and thalami; WM, white matter; n/a, sample was not available; F, favorable; U, unfavorable; y, yes; n, no.*.

### Perinatal Characteristics and NfL Levels

Median (IQR) age at obtaining the first sample in the mild HIE group was 24 (13, 34) h (Table 3). Three of the 11 babies in this group had a detectable or raised NfL level. Median (IQR) NfL levels in this whole group were 0 (0, 41) pg/mL. In the babies who had received TH (*n* = 26), there was no statistically significant correlation between the highest NfL level and 10-min Apgar, worst pH, and base deficit in the first hour. Similarly, there was no significant correlation between the presence of electrographic seizures on amplitude-integrated EEG monitoring and NfL levels.

**Table 3:** The median neurofilament light levels and age after birth of patients in the study.

	S1 (pg/mL)	Age (h)	S2 (pg/mL)	Age (h)	S3 (pg/mL)	Age (h)
MildNo TH	0 (0, 41)	24 (13, 34)	–	–	–	–
Mod-severeTH favorable	0 (0, 110)	16 (11, 22)	71 (0, 200)	57 (48, 62)	164 (47, 327)	103 (96, 108)
Mod-severeTH unfavorable	173 (29, 681)	18 (15, 24)	381 (180, 968)	54 (42, 60)	844 (511, 1,014)	96 (95, 104)

*Interquartile ranges are expressed in brackets*.

*TH, therapeutic hypothermia*.

### Plasma NfL Levels in Babies with Unfavorable Outcome Compared to the Other Groups

**Figure 2:**
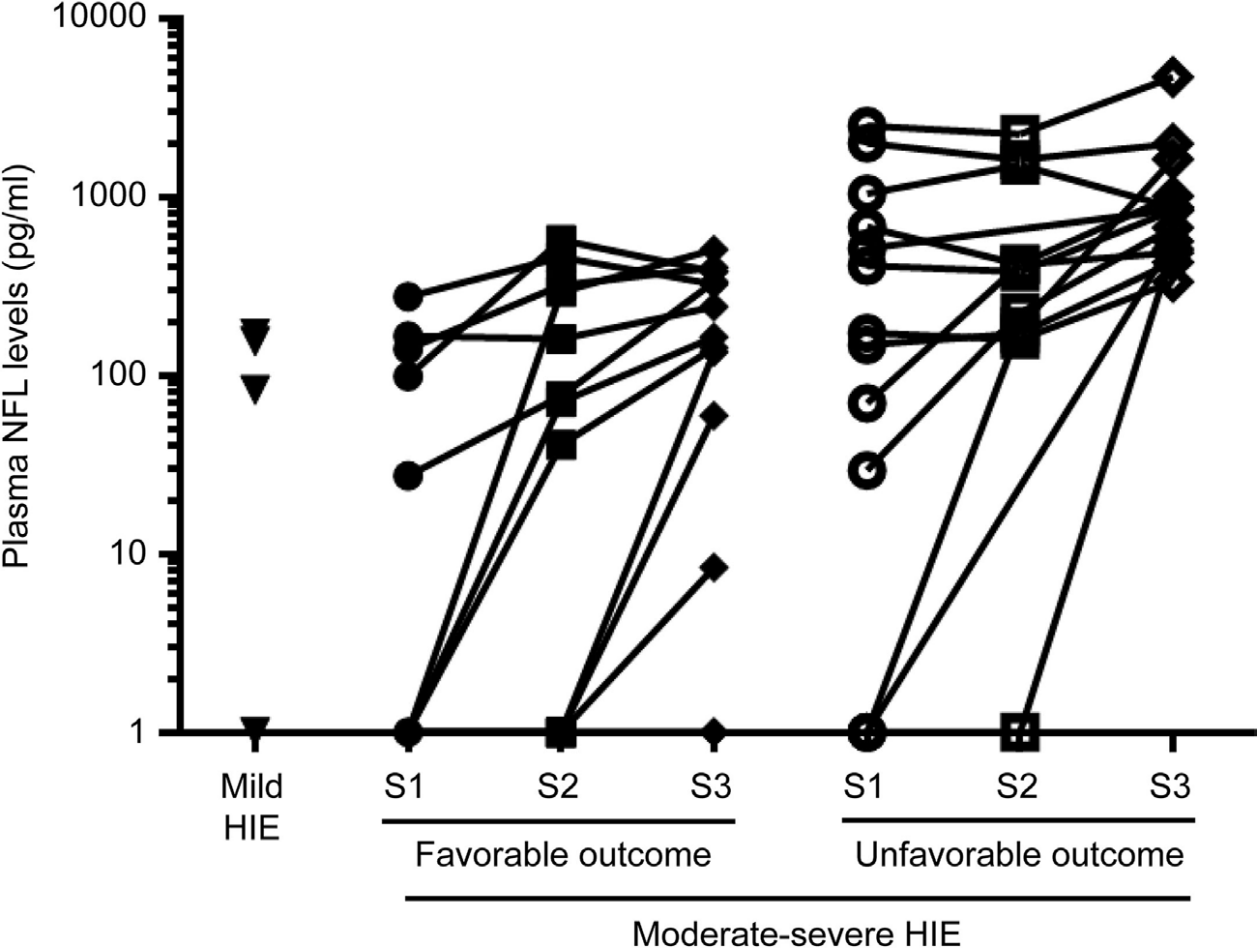
Evolution of neurofilament light (NfL) over the three time points in the therapeutic hypothermia groups.

There was no significant difference between groups in age at obtaining samples S1, S2, and S3 (Table 3). Median values (IQR) of the three groups at the different time points are shown in Table 3. The evolution of NfL levels during cooling for individual babies is shown in Figure 2. After log transformation and analysis of variance, the difference in initial NfL levels between the three groups was highly significant (*F* = 25.83, *p* < 0.0001).

### Temporal Increase in Plasma NfL Level in Babies Treated with TH

**Figure 3:**
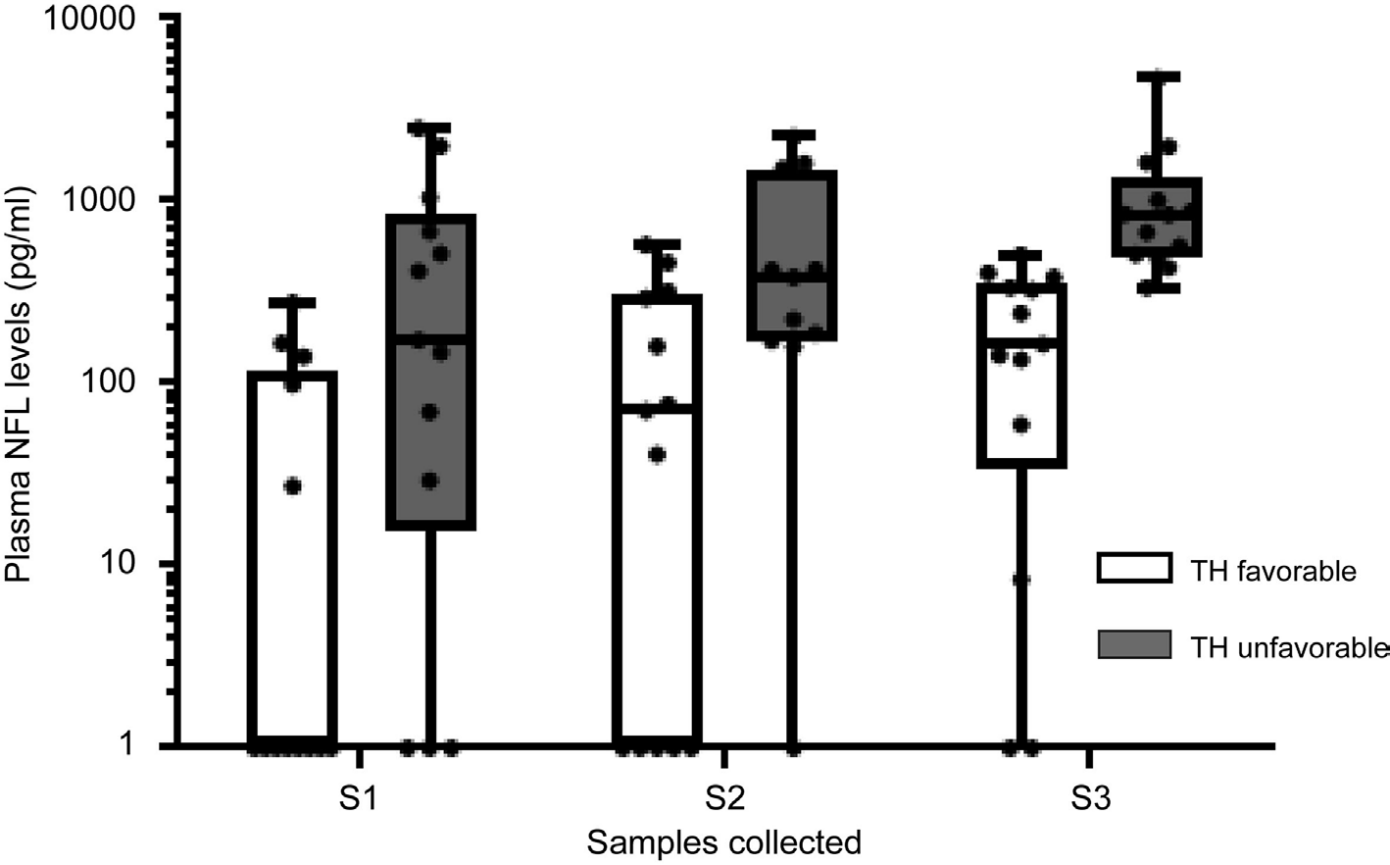
Temporal changes in plasma neurofilament light (NfL) levels in groups with different severity of brain injury. Data expressed as median with interquartile ranges and whiskers with minimum to maximum.

For the babies who underwent TH, sequential NfL levels were higher in those with MR-evident brain injury predictive of unfavorable outcome compared to NfL levels in those with MRI predictive of favorable outcomes (mixed models, two-way ANOVA, *F* = 27.6, *p* < 0.001) (Figure 3). In both groups, median NfL levels increased over the course of TH treatment; initial NfL levels (S1) were not only higher in babies with unfavorable outcomes but also remained higher throughout the time course, in comparison to those from babies with favorable outcomes.

All infants with unfavorable outcomes had some rise in NfL levels. All infants with NfL levels >400 pg/mL at the first sample (*n* = 6) had unfavorable outcomes. Of the 13 infants with favorable outcomes, 10 had some rise in NfL levels. Of these 10, four had completely normal MR imaging.

Six infants from the whole TH group with raised NfL levels in the first sample after reaching target hypothermia temperature (S1) showed a decrease in levels by the time of the second sample prior to commencing rewarming (S2). Yet all six showed a subsequent rise in NfL levels by the time of the third sample (post rewarming, S3).

### Diagnostic Accuracy of Plasma NfL Levels in Prediction of Unfavorable Outcome

**Figure 4:**
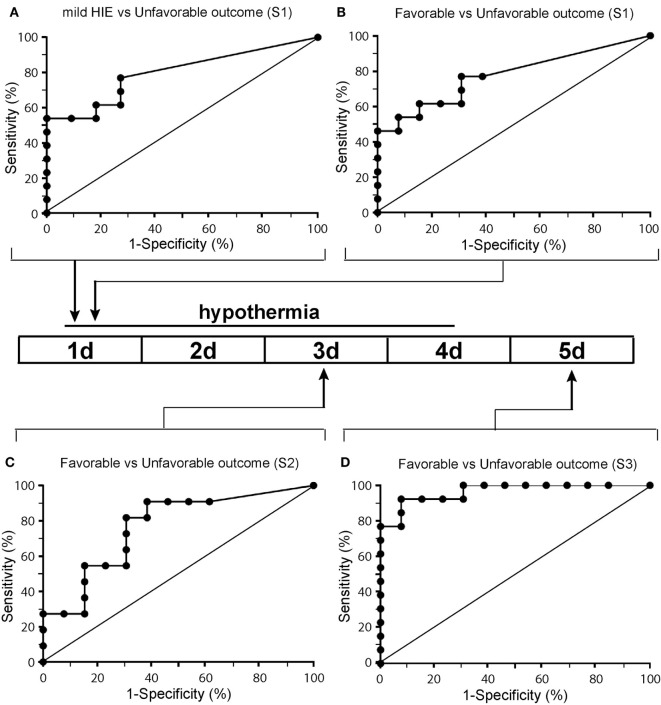
Receiver operated characteristics curves of the neurofilament light (NfL) biomarker in the plasma of newborns with panel **(A)** representing a comparison of NfL level for babies with mild hypoxic-ischemic encephalopathy (HIE) [no therapeutic hypothermia (TH)] with TH babies with unfavorable outcome for the first sample and panels **(B–D)** representing a comparison of babies who had undergone TH at samples 1 **(B)**, sample 2 **(C)**, and sample 3 **(D)**.

ROCs were performed to evaluate the diagnostic value of plasma NfL levels in predicting MRI predictive of abnormal outcome in newborns with HIE. At all three sampling time points, NfL cut-off levels likely to be predictive of unfavorable outcomes were obtained (Figure 4; Table 3). In babies undergoing TH, an NfL level of >29 pg/ml at S1 was predictive of unfavorable outcome, with area under the curve (AUC) of 0.78, *p* = 0.017, sensitivity 77%, and specificity 69% (Table 4). For S3, an NfL level of >417 pg/mL was strongly predictive of unfavorable outcome with AUC 0.97, *p* < 0.0001, sensitivity 92%, and specificity 92%. On arranging the subjects in rank order of NfL levels at S3, it is notable that babies with higher NfL levels were more likely to have cerebral abnormalities on MRI (Table 2). However, one newborn with an NFL level >417 pg/mL had MRI predictive of favorable outcome and two with NFL levels <417 pg/mL had MRI predictive of unfavorable outcomes.

**Table 4:** Diagnostic power of neurofilament light levels comparing babies newborns with favorable and unfavorable outcomes within the therapeutic hypothermia group at samples S1, S2, and S3.

Sample	Area under curve	SE	*p* Value	Cutoff (pg/ml)	Sensitivity (%)	Specificity (%)	PPV (%)	NPV (%)
S1	0.775	0.094	0.017	29	77	69	67	73
S2	0.766	0.100	0.028	166	82	69	69	82
S3	0.965	0.032	<0.0001	417	92	92	92	86

*PPV, positive predictive value; NPV, negative predictive value*.

## Discussion

We demonstrate that it is feasible to study NfL levels in plasma from newborn babies. In newborns undergoing TH for HIE, NfL levels were raised as early as 24 h after birth in those who had cerebral MRI predictive of unfavorable neurodevelopmental outcome. NfL levels rose during the course of TH and were highest post rewarming. A cutoff NfL level >417 pg/mL after rewarming was strongly predictive of unfavorable outcome. Thus, raised NfL levels may be an important biomarker of neuronal, and more specifically axonal damage, in this group of babies.

Although safe, TH does not benefit all babies with HIE (3, 4). Long-term follow-up from randomized controlled trials shows that up to 50% of babies who underwent TH nevertheless had adverse outcomes of death and disability (5, 6). At present, there are no clinically established biomarkers of brain injury available at the cot side that allow objective selection of babies for existing neuroprotective strategies such as TH or for stratifying babies for additional, novel interventions. The data from our study are consistent with the preliminary findings from Toorell et al. (15) and support plasma NfL as being a suitable candidate early biomarker worthy of further investigation. Furthermore, serum NfL levels have been shown to be raised in adult traumatic brain injury (18, 19).

In their study, Toorell et al. (15) compared NfL levels from the cord blood of 10 asphyxiated children with that of cord blood from 18 controls. Of their 10 cases, seven did not suffer HIE. In our study, all cases undergoing TH had fulfilled local criteria for treatment. Although in this work we did not study cord blood, we were able to study the temporal evolution of the plasma NfL levels through the TH process and relate the NfL levels to MRI outcome. In this study, we did not carry out a comparison of a perinatal severity of “sickness” scoring system to NfL levels.

We noted that half the babies in the TH group had raised NfL levels in the first specimen. We speculate that the insult may have occurred some time prior to delivery in some of this group. In six of all the babies who received TH with raised NfL levels in the first specimen, there was a drop in NfL level noted in the second sample with the baby still being cooled. However, in all six, a rise in NfL levels was noted after rewarming was complete. This may be an observed effect of TH; a better understanding of the temporal changes in plasma NfL levels may also allow for more precise timing of the occurrence of the hypoxic-ischemic insult as well as better monitoring of the response to neuroprotective treatments.

To date, various blood-detected molecules have been proposed as potential biomarkers of brain injury after HIE (8). However, many of these markers are not specific to brain injury. One potential advantage of neurofilaments as biomarkers in the newborn is that they are neuron-specific cytoskeletal proteins, released into the CSF and blood when neurons are damaged (10). In this work, we have investigated only the NfL subunit because researchers from our institution and others have shown that NfL levels are raised in neurologic conditions in which neuronal and particularly axonal injury is important (11–13). Further work is required to test the utility of the other neurofilament subunits as potential biomarkers.

We used a commercially available sandwich ELISA kit that uses a heterophilic antibody designed for detecting NfL levels in the CSF, with a lower detection limit of 32 pg/mL. At present, this test can be carried out and results obtained within 3 h. We were able to show a significant difference between the groups in relation to cerebral injury on MRI, as well as a function of time. This antibody has been used in other studies on plasma and serum, albeit with other platforms (12). The newborns with unfavorable outcomes in this study had NfL levels (median 792 pg/mL) that were higher than those observed in the plasma from adults with ALS (median 96 pg/mL) (11). We postulate that this difference relates to the time course of the insult in newborns being more acute and the nature of the insult more global, compared to the more focal and chronic, slow-developing nature of the pathology in ALS.

A limitation of our study is that we did not have plasma specimens from cord blood, or from the babies before cooling was commenced. Also, we do not have samples from infants with moderate–severe HIE who did not receive TH. Although longer term neurodevelopmental outcomes are as yet unavailable, the method of scoring the MRIs that we used has been shown to be predictive of longer term outcomes (17).

## Conclusion

Our data suggest that plasma NfL is a potentially useful biomarker for predicting unfavorable MRI outcomes in newborns with moderate–severe HIE undergoing TH. As a biomarker at 24 h of age and beyond, NfL levels may be of value for adjunctive neuroprotective treatments targeting the reperfusion and “repair” phases after hypoxia-ischemia. A larger study is required to confirm our findings, and should include plasma specimens from within the first 6 h after birth and explore the potential additional utility of intermediate and heavy subunits of neurofilaments. Further work is also required to correlate NfL levels with longer term neurodevelopmental outcomes.

## What is Known About This Topic?

– Therapeutic hypothermia does not benefit all newborns after hypoxic-ischemic encephalopathy
– There are no blood biomarkers that allow selection of babies for neuroprotection at present
– Neurofilament light (NfL) chains have been shown to be raised in blood and CSF from patients with other neurological pathology as well as cord blood from babies with asphyxia

## What This Study Adds

– It is feasible to measure NfL levels, a marker of axonal injury, in the plasma of newborns
– NfL levels are raised at 24 h in newborns undergoing therapeutic hypothermia with cerebral tissue injury predictive of unfavorable outcome
– NfL levels may allow stratification of newborns to adjunctive brain saving treatments

## Ethics Statement

This study was carried out in accordance with the recommendations of Bromley Research Ethics Committee with written informed consent from all parents. All parents gave written informed consent in accordance with the Declaration of Helsinki. The protocol was approved by the Bromley Research Ethics Committee.

## Author Contributions

DS conceptualized and designed the study, designed the data collection sheets, recruited patients for the study, performed the data analysis, drafted the initial manuscript, and approved the final manuscript as submitted. DS, the principal investigator, had full access to all the data in the study and took responsibility for the integrity of the data and the accuracy of the data analysis. VP assisted with designing the data collection sheets, recruited patients for the study, assisted with obtaining tissue samples and collecting and managing the data, assisted in drafting the manuscript, and approved the final manuscript as submitted. JE reviewed the MR images, assisted in preparing the manuscript, and approved the final manuscript. OK recruited patients for the study, reviewed the MR images, assisted in preparing the manuscript, and approved the final manuscript. GE recruited patients for the study, assisted with obtaining tissue samples and data collection, assisted in preparing the manuscript, and approved the final manuscript. NG recruited patients for the study, assisted with obtaining tissue samples and data collection, assisted in preparing the manuscript, and approved the final manuscript. PC assisted with designing the data collection sheets, recruited patients for the study, assisted with obtaining tissue samples and data collection, assisted in preparing the manuscript, and approved the final manuscript. AM-T provided intellectual input, assisted in preparing the manuscript and approved of the final manuscript. PY conceptualized and designed the study, performed the laboratory analysis, assisted with the data analysis, assisted in preparing the manuscript, and approved the final manuscript.

## Conflict of Interest Statement

The authors declare that the research was conducted in the absence of any commercial or financial relationships that could be construed as a potential conflict of interest. The reviewer RC, RF and handling Editor declared their shared affiliation.
