# Corrigendum: Raised plasma neurofilament light protein levels are associated with abnormal MRI outcomes in newborns undergoing therapeutic hypothermia

**DOI:** 10.3389/fneur.2024.1474489

**Published:** 2024-09-30

**Authors:** Divyen K. Shah, Vennila Ponnusamy, Jane Evanson, Olga Kapellou, Georgia Ekitzidou, Neelam Gupta, Paul Clarke, Adina T. Michael-Titus, Ping K. Yip

**Affiliations:** ^1^The Royal London Hospital, Barts Health NHS Trust, London, United Kingdom; ^2^The Centre for Neuroscience and Trauma, Barts and The London School of Medicine and Dentistry, Blizard Institute, Queen Mary University of London, London, United Kingdom; ^3^Centre for Genomics and Child Health, Barts and The London School of Medicine and Dentistry, Blizard Institute, Queen Mary University of London, London, United Kingdom; ^4^Ashford and St. Peter's Hospitals NHS Foundation Trust, Chertsey, United Kingdom; ^5^Homerton University Hospital NHS Foundation Trust, London, United Kingdom; ^6^University Hospital Southampton, Southampton, United Kingdom; ^7^Norfolk and Norwich University Hospitals NHS Foundation Trust, Norwich, United Kingdom

**Keywords:** neurofilament proteins, hypoxic-ischemic encephalopathy, therapeutic hypothermia, MRI imaging, neuroprotection, biomarkers

In the published article, there was an error in Table 2 as published. For patients 1 and 3, the cell for the column entitled “Neurologic exam prior to discharge” should read “Severe, died after 18 months” instead of “Severe, died.” The corrected Table 2 and its caption “Patients arranged in descending rank order of neurofilament light levels at S3 in relation to MRI findings and neurological exam at discharge. The latter is classified primarily in terms of abnormality of tone” appear below.

**Table 2:** Patients arranged in descending rank order of neurofilament light levels at S3 in relation to MRI findings and neurological exam at discharge. The latter is classified primarily in terms of abnormality of tone.

	**S1 (pg/mL)**	**S2 (pg/mL)**	**S3 (pg/mL)**	**Age at peak (h)**	**Electrical seizures (Y/N)**	**PLIC**	**BGT**	**WM**	**Cortex**	**Composite MRI Group**	**Sentinel event (Y/N)**	**Comment**	**Neurologic exam prior to discharge**
1	2,506	2,255	4,698	100	Y	Loss	Severe	Moderate	Normal	U	N		Severe, died after 18 months
2	1,999	1,615	1,993	18	N	Equivocal	Normal	Severe	Moderate	U	Y	Cord prolapse	Mild
3	0	189	1,626	124	Y	Loss	Severe	Severe	Severe	U	N		Severe, died after 18 months
4	70	423	1,014	114	N	Loss	Severe	Mild	Mild	U	Y	Breech, head entrapment	Moderate, tube feeds
5	1,046	1,512	874	31	Y	Normal	Moderate	Normal	Normal	U	N		Normal
6	516	n/a	849	96	N	Equivocal	Normal	Severe	Severe	U	Y	Placental abruption	Severe, tube feeds
7	413	381	844	118	Y	Normal	Moderate	Severe	Moderate	U	N		Moderate, tube feeds
8	29	225	674	95	N	Loss	Severe	Severe	Severe	U	Y	Shoulder dystocia	Severe, tube feeds
9	0	n/a	563	94	Y	Equivocal	Moderate	Mild	Moderate	U	N		Moderate, tube feeds
10	0	0	511	96	n/a	Loss	Moderate	Mild	Normal	U	N		Severe, tube feeds
11	0	294	506	100	Y	Normal	Mild	Mild	Normal	F	N		Normal
12	681	419	490	18	N	Loss	Severe	Normal	Moderate	U	N		Normal
13	141	321	403	115	N	Normal	Normal	Normal	Normal	F	Y	Placental abruption	Normal
14	99	577	381	67	N	Normal	Normal	Normal	Normal	F	Y	Head entrapment	Normal
15	28	77	336	101	N	Normal	Normal	Mild	Mild	F	Y	Shoulder dystocia	Normal
16	173	159	334	101	N	Loss	Severe	Moderate	Mild	U	N		Normal
17	277	457	324	62	N	Normal	Normal	Normal	Normal	F	N		Mild
18	166	160	242	86	Y	Normal	Mild	Normal	Normal	F	N		Normal
19	0	71	164	108	n/a	Normal	Normal	Mild	Normal	F	N		Normal
20	148	172	151	48	Y	Loss	Moderate	Severe	Normal	U	N		Moderate, tube feeds
21	0	41	143	115	n/a	Normal	Normal	Normal	Normal	F	N		Normal
22	0	0	136	114	Y	Normal	Normal	Moderate	Normal	F	N		Normal
23	0	0	59	102	N	Normal	Normal	Normal	Normal	F	N		Normal
24	0	0	8	-	n/a	Normal	Normal	Normal	Normal	F	N		Normal
25	0	0	0	-	Y	Normal	Normal	Mild	Mild	F	N		Normal
26	0	0	0	-	N	Normal	Normal	Normal	Normal	F	Y	Uterine rupture	Normal

PLIC, posterior limb of internal capsule; BGT, basal ganglia and thalami; WM, white matter; n/a, sample not available; F, favorable; U, unfavorable; y, yes; n, no.

The authors apologize for this error and state that this does not change the scientific conclusions of the article in any way. The original article has been updated.

